# Plasma membrane proteomic analysis by TMT-PRM provides insight into mechanisms of aluminum resistance in tamba black soybean roots tips

**DOI:** 10.7717/peerj.9312

**Published:** 2020-06-10

**Authors:** Yunmin Wei, Caode Jiang, Rongrong Han, Yonghong Xie, Lusheng Liu, Yongxiong Yu

**Affiliations:** Southwest University, College of Animal Science and Technology, Chongqing, China

**Keywords:** Aluminum toxicity, Tamba black soybean, Plasma membrane proteins, Proteomics, TMT, PRM

## Abstract

Aluminum (Al) toxicity in acid soil is a worldwide agricultural problem that inhibits crop growth and productivity. However, the signal pathways associated with Al tolerance in plants remain largely unclear. In this study, tandem mass tag (TMT)-based quantitative proteomic methods were used to identify the differentially expressed plasma membrane (PM) proteins in Tamba black soybean (TBS) root tips under Al stress. Data are available via ProteomeXchange with identifier PXD017160. In addition, parallel reaction monitoring (PRM) was used to verify the protein quantitative data. The results showed that 907 PM proteins were identified in Al-treated plants. Among them, compared to untreated plants, 90 proteins were differentially expressed (DEPs) with 46 up-regulated and 44 down-regulated (fold change > 1.3 or < 0.77, *p* < 0.05). Functional enrichment based on GO, KEGG and protein domain revealed that the DEPs were associated with membrane trafficking and transporters, modifying cell wall composition, defense response and signal transduction. In conclusion, our results highlight the involvement of GmMATE13, GmMATE75, GmMATE87 and H^+^-ATPase in Al-induced citrate secretion in PM of TBS roots, and ABC transporters and Ca^2+^ have been implicated in internal detoxification and signaling of Al, respectively. Importantly, our data provides six receptor-like protein kinases (RLKs) as candidate proteins for further investigating Al signal transmembrane mechanisms.

## Introduction

Al is the most abundant metallic element in the earth’s crust, and it is normally present as aluminosilicates and oxides with nonphytotoxicity (Sade et al., 2016). At pH < 5 in acid soil, Al can be released into the soil in the form of soluble Al^3+^ ion, which damages the root cell and further influences the uptake of nutrients and water, consequently resulting in severe loss of plant yield (Kochian et al., 2015). It has been well established that Al-tolerant plants in acid soils have developed both internal tolerance and external exclusion strategies in resistance to Al toxicity (Jiang et al., 2018). The former involves Al^3+^ influx across the root cell plasma membrane into the cytosol, where Al^3+^ is detoxified by forming nonphytotoxic organic acid (OA)-Al chelates, which are eventually sequestered into the vacuoles via multifunctional tonoplast transporters (Singh et al., 2017; Zhang et al., 2019). The latter links Al-induced root exudation of OA into the rhizosphere, also forming OA-Al complexes and thereby preventing Al^3+^ to enter into the root. Therefore, comprehensive understanding of Al-resistant genes will facilitate the development of plant varieties suitable for cultivation in acidic soils (Li et al., 2014; Poschenrieder, Busoms & Barcelo, 2019).

In recent decades, a number of genes and pathways responsible for Al-induced exudation of OAs have been identified, which include *ALMTs* encoding malate efflux transporters and *MATEs* encoding multidrug and toxic compound extrusion proteins (Sade et al., 2016; Zhang et al., 2019). Internally, *Nrat1* encoded Nramp aluminum transporter 1, which acts in concert with a tonoplast-localized half-size ABC transporter encoded by *ALS1*, to sequester Al^3+^ into the vacuole in roots of rice (Huang et al., 2012; Liu, Pineros & Kochian, 2014). Transcription factors (TFs) essential for external and internal detoxification of Al^3+^ have also been identified. These TFs include STOP1 (sensitive to proton rhizotoxicity 1) in *Arabidopsis* (Iuchi et al., 2007), ART1 (Al-resistant transcription factor 1) in rice (Yamaji et al., 2009) and NAR1 (NAC-type transcription factor) in rice bean (Lou et al., 2020). In stylo roots, the signaling cascades of Al-induced citrate exudation comprise heterotrimeric G-proteins, phospholipase C (PLC), inositol triphosphate (IP3), diacylglycerol (DAG), Ca^2+^ and protein kinases (PK) (Jiang et al., 2018). Proteins associated with signaling and vacuolar sequestration of Al were also documented in rice (Wang et al., 2013; Yang et al., 2013). Despite these advance, current researches mainly focus on the signaling pathways after Al signal transmembrane; the mechanisms underlying Al signal transmembrane remain to be clarified.

The plasma membrane (PM) acts as the primary interface of nutrient and information exchange between the cellular cytoplasm and the extracellular environment, with membrane-associated proteins functioning as key effectors of biological processes, such as plant growth, development and in respond to a myriad of environmental cues (Yadeta, Elmore & Coaker, 2013; Aloui et al., 2018). The PM has been regarded as the first target for Al^3+^, due to its strong binding to phospholipids (Ahn & Matsumoto, 2014). These evidence enable us to suppose that transmembrane of Al signal is mediated by cell membrane.

Transcriptome profiling on Al-stressed plants, including *Arabidopsis*, soybean, rice and others, have disclosed robust gene transcriptional changes related to signal transduction, TFs, transporters, oxidative stress pathway and energy metabolism (Jiang et al., 2018; Zhang et al., 2019). Nevertheless, the changes between the abundance of mRNA and its encoded protein is not always consistence (Zheng et al., 2014). In particular, biological processes are ultimately embodied by proteins, hence proteomic profiling is critical for offering an accurate protein changes triggered by Al stress as demonstrated in soybean (Huang et al., 2017), rice (Wang et al., 2013; Yang et al., 2013), wheat (Oh et al., 2014) and maize (Requejo & Tena, 2005).

TBS is an Al-tolerant genotype due to its great potential for Al^3+^ tolerance via secretion of citrate under Al stress (Wu et al., 2012). However, there are limited reports about proteins response to Al stress in TBS. PM proteomics of TBS provide us information that suggests us a number of associations of the PM proteins and Al toxicity and resistance mechanism. Hence, the PM proteomic changes under Al stress in TBS roots were detected by a combination of TMT labeling and HPLC-MS/MS analysis in this study. In addition, PM proteins play important roles in Al signal transmembrane were presented (Li et al., 2015). The identified Al-induced changes of PM proteins lay a foundation for further study of the transmembrane mechanism of aluminum signaling related to citric acid secretion from TBS roots.

## Materials and Methods

### Plant culture and measurements of Al^3+^ treatments

Seeds of TBS were rinsed soaked in double distilled H_2_O for 30 min and then transferred to moist towel in the dark at 25 °C. After germination, uniform seedlings were transferred onto a floating mesh in opaque beaker with 1/2 strength hoagland nutrient solution. The nutrient solution was renewed every day. Plants were cultured in a tissue culture room at 27/22 °C day/night temperatures with 14 h of light (200 µmol photons m^−2^·s^−1^). When the true leaf was fully expanded, the seedlings were pre-treated with 0.5 mM CaCl_2_ (pH 4.3) for 24 h. To choose optimum treatment concentration of AlCl_3_, the roots were then transferred into 0.5 mM CaCl_2_ solution containing 0, 25, 50, 75, 100 µM AlCl_3_ at pH 4.3 for 24 h. Furthermore, to choose optimum time, the pre-treated roots were transferred to 0.5 mM CaCl_2_ (pH 4.3) containing 50 µM AlCl_3_ for 1, 2, 3, 4, 5, 6 d, respectively. After treatments, the relative growth rate (RRG) (Jiang et al., 2018), Evans blue staining (Arroyave et al., 2013) and Chrome Azurol S staining (Jiang et al., 2018) methods were used to measure the effects of Al treatment, root exudates were collected and concentrated for citrate content by enzymic determination (Zhao et al., 2003). Each experiment was repeated for five times. Two-week-old seedlings were transferred into 0.5 mM CaCl_2_ solution containing AlCl_3_ at 0 (CK), 50 µM (Al treatment) at pH 4.3 for 3 d. After treatment, root apices (0–2 cm) were excised, immediately frozen in liquid nitrogen and then harvested for isolating total RNA and the PM proteins.

### Plasma membrane protein enrichment

The plasma membrane proteins were extracted as reported (Voothuluru et al., 2016). Briefly, the frozen root tissues were taken out from liquid nitrogen and quickly ground into fine powder. After adding ice-cold buffer H (330 mM sucrose, 50 mM Na_4_P_2_O_7_, 25 mM NaF, 5% glycerol, 0.5% PVP, 10 mM EDTA, 1 mM Na_2_MoO_4_, 0.1% protease inhibitor cocktail, 3 mM DTT) (1 mL per g fresh weight of tissue), the samples were centrifuged for 10 min (10,000×*g* 4 °C). Next, we collected the supernatant and centrifuged for 30 min (100,000×*g* at 4 °C). The obtained crude microsomal pellets were washed with buffer H without DTT, and incubated in buffer H with 0.02% Brij-58 but without DTT (2 μL/μg of crude microsomal protein on ice) for 45 min to dissolve. The PM-enriched protein fractions were obtained after centrifuged for 30 min (100,000×*g* at 4 °C). The protein concentration was measured by BCA kit (Beyotime Biotechnology, Beijing, China) according to the manufacturer’s instructions.

### TMT proteomics analysis

According to Guo et al. (2019), protein samples were subjected to trypsin digestion and TMT Labeling, then the tryptic peptides were fractionated into fractions by high pH reverse-phase HPLC and subjected to NSI source followed by tandem mass spectrometry (MS/MS) in Q Exactive TM Plus (Thermo, Waltham, MA, USA) coupled online to the UPLC. The resulting MS/MS data were processed using Maxquant search engine (v.1.5.2.8). Spectra search was performed against *Phytozome 12* (the plant genomics resource, *Glycine ma*x Wm82.a2.v1; https://phytozome.jgi.doe.gov/pz/portal.html#!info?alias=Org_Gmax) concatenated with reverse decoy database. FDR of protein identification and PSM identification was set to 1%. To obtain PM proteins for analysis, subcellular localization was performed by wolfpsort (http://www.genscript.com/psort/wolf_psort.html). For protein abundance ratios measured using TMT data, we considered a 1.3-fold change at *p* < 0.05 as the thresholds for identifying significant changes.

### Bioinformatics analysis

Functional annotation of PM proteins was performed using the GO annotation (http://www.ebi.ac.uk/GOA/) and protein domain annotation (http://www.ebi.ac.uk/interpro/). To classify and group the identified proteins, the KEGG pathway (http://www.genome.jp/kegg/) was analyzed. Among GO annotation, GO annotation proteome was derived from the UniProt-GOA database. Firstly, Converting identified protein ID to UniProt ID and then mapping to GO IDs by protein ID. If some identified proteins were not annotated by UniProt-GOA database, the InterProScan soft would be used to annotated protein’s GO functional based on protein sequence alignment method. Then proteins were divided into biological process and molecular function. To classify and group the identified proteins, KEGG (http://www.genome.jp/kegg/) database was used to annotate protein pathway. Firstly, using KEGG online service tools KAAS to annotated protein’s KEGG database description. Then mapping the annotation result on the KEGG pathway database using KEGG online service tools KEGG mapper. Based on GO, KEGG pathway and protein domain, enrichment analysis of differentially expressed proteins were performed. For each functional enrichment, a two-tailed Fisher’s exact test was used to test the enrichment of differentially expressed proteins relative to all identified PM proteins. GO, KEGG pathway and protein domain with a *p* < 0.05 were considered significant.

### Targeted protein quantification by parallel reaction monitoring

To confirm TMT data, twenty proteins were randomly chosen and quantified by PRM-MS analysis. According to the TMT data, signature peptides for the target proteins were defined and only unique peptide sequences were determined for PRM analysis. As in the TMT experiment, protein preparation and trypsin digestion were performed. After HPLC-MS/MS Analysis, data of each sample was collected using the PRM acquisition method to quantify the targeted proteins abundance. Three biological replicates of each group were performed. The PRM-MS analysis in our study is supported by Jingjie PTM BioLabs (Hangzhou, China).

### cDNA synthesis and RT-qPCR analysis

Total RNA was isolated using the RNAiso Plus kit (Takara, Dalian, China) according to the manufacturer’s description. A Nano Drop 2000 spectrophotometer (Thermo, Waltham, MA, USA) was used to measure concentration of total RNA. After DNase treatment, 1 µg total RNA was used as template for each cDNA synthesis by using PrimeScript RT reagent kit (Takara, Dalian, China). Real-time fluorescent quantitative PCR (RT-qPCR) was performed according to our previous work (Jiang et al., 2018). *18s rRNA* gene (M16859) was used as endogenous control for RT-qPCR. Primers used were given in Table S1.

### Statistical analyses

Statistical analysis was performed using one-way ANOVA or two-sided Student’s *t*-test. *p* < 0.05 was considered as of significant difference. Origin Pro (Version 8.5) was used to prepare graphs.

## Results

### Effect of Al^3+^ treatment on citrate secretion of TBS roots

**Figure 1:**
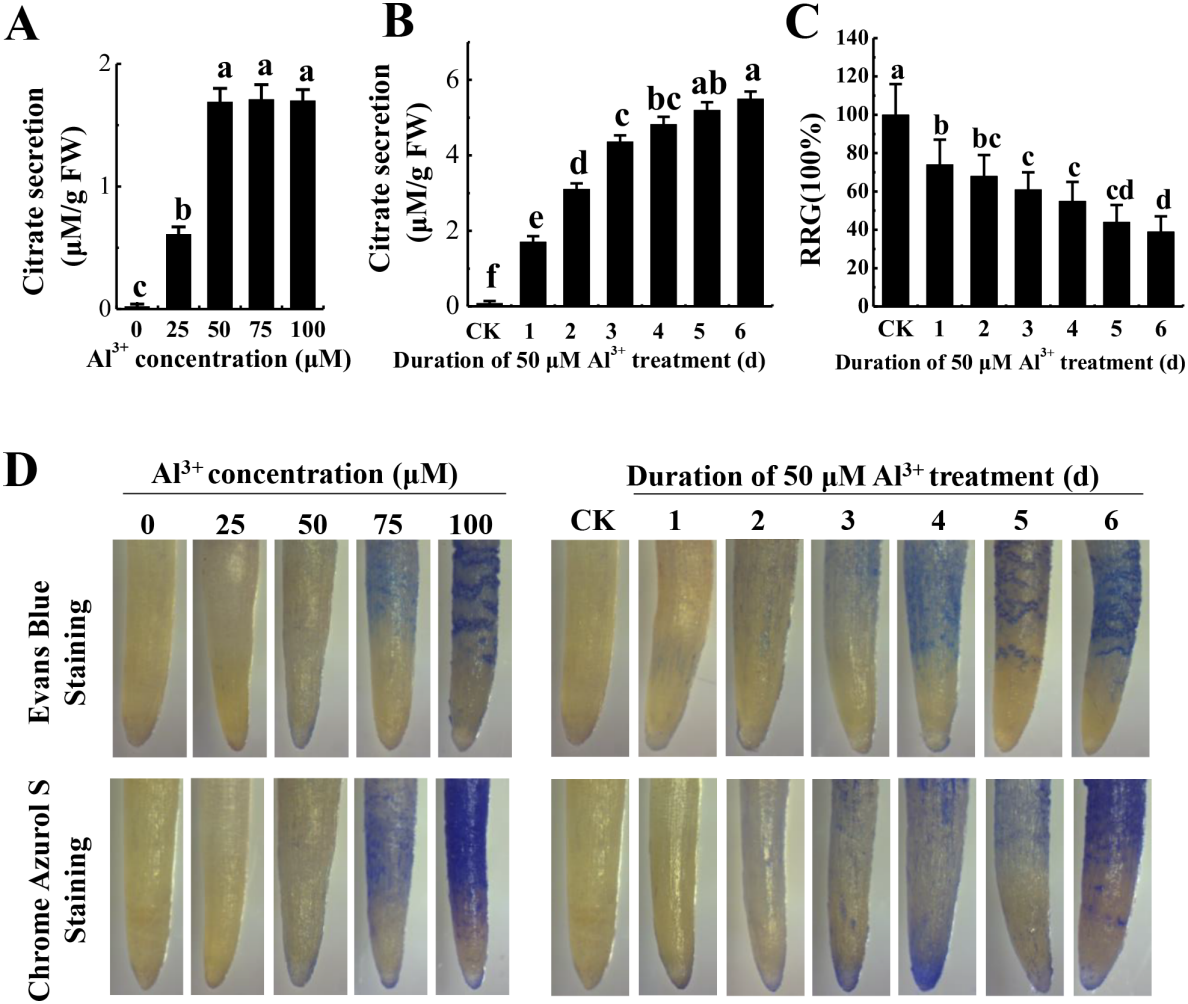
Effect of Al^3+^ treatment on seedling roots of TBS. (A) Citrate secretion of roots treated with various Al^3+^ concentration for 24 h. (B) Citrate secretion of roots treated with 50 µM Al^3+^ during different treatment time. (C) Effects on the RRG of TBS roots treated with 50 µM Al^3+^ during different treatment time. (D) Evans Blue and Chrome Azurol S staining for roots of (A) and (B) treatment. Values are represent as Mean ± SE (*n* = 5). Different letters above columns indicate significance at *p* < 0.05. CK, the control without Al^3+^ treatment (pH 4.3) for 3 d.

To optimize Al^3+^ treatment for proteome analysis, we measured citrate secretion at different concentration and duration of Al^3+^ treatment. The results showed that citrate secretion increased with the increase of AlCl_3_ concentration and reached the maximum at 50 μM AlCl_3_ with no apparent damage to the roots as shown by Evans blue and Chrome Azurol S staining (Fig. 1A). Then, citrate secretion was detected after different time of 50 μM AlCl_3_ treatment. Although citrate secretion was increased with the prolonging of treatment time, a sharp enhancement of citrate secretion was detected at the third day with no more than a 25% inhibition of relative root growth (RRG) compared to the control (−Al^3+^) (Figs. 1B and 1C). Moreover, much lower of Evans blue and Chrome Azurol S staining was detected in the group treated with 50 μM AlCl_3_ for 3 d than that in the groups with 50 μM AlCl_3_ treatment for 4–6 d (Fig. 1D). Therefore, 50 μM AlCl_3_ for 3 d was chose for further experiments.

### Proteome profiles in TBS roots after Al^3+^ treatment

**Figure 2:**
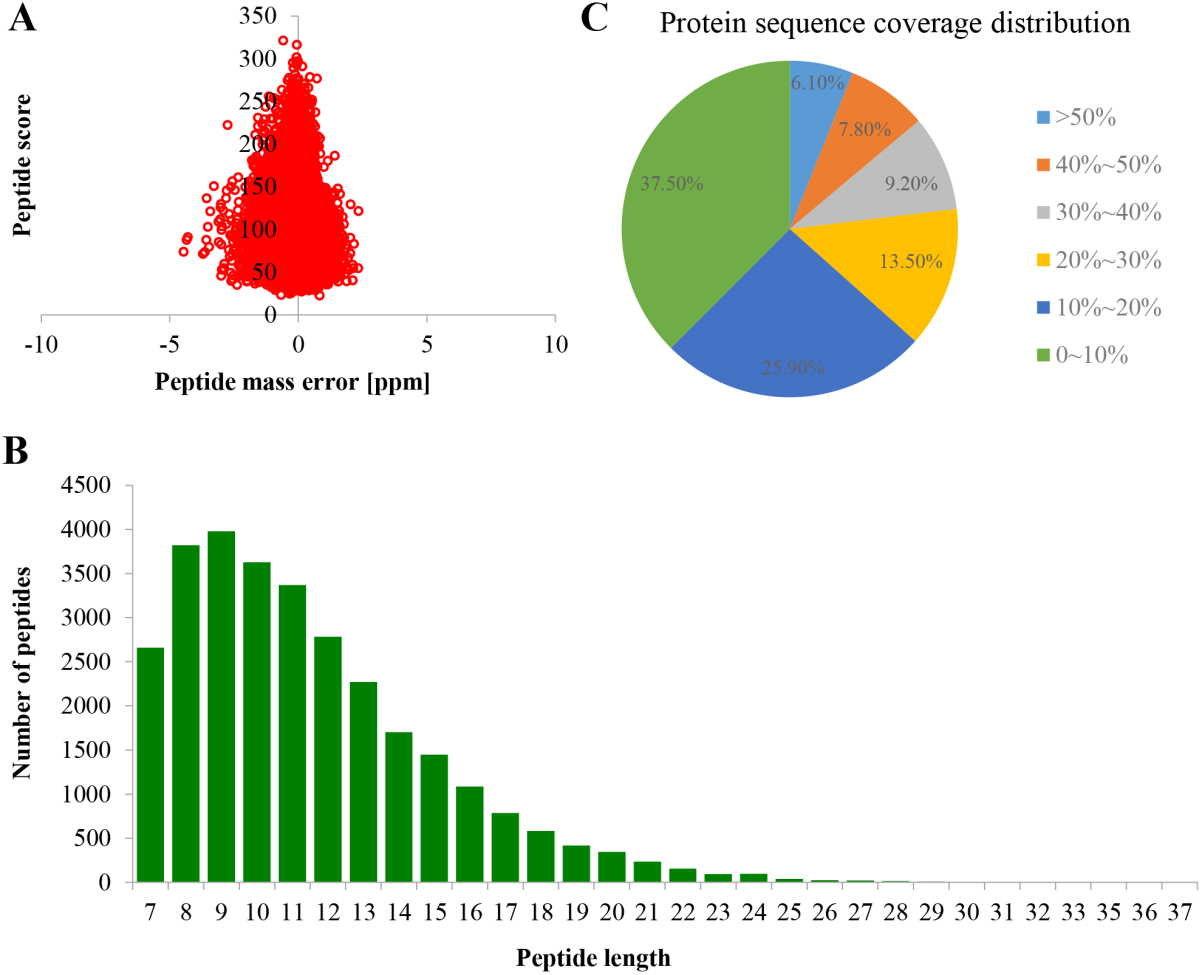
Quality control validation of mass spectrometer (MS) data. (A) Volcano map of error rate distribution by mass spectrometry. (B) Length distribution of peptides. (C) Distribution of the proteins’ sequence coverage. Three biological replicates were performed.

Proteome profiles were evaluated using TMT labeling and HPLC-MS/MS techniques in the PM of TBS seedlings after treatment with 0 and 50 μM AlCl_3_ for 3 d (Fig. S1). The mass spectrometry proteomics data have been deposited to the ProteomeXchange Consortium via the PRIDE partner repository with the dataset identifier PXD017160. A total of 6,877 proteins were obtained, of which 5,524 were available for quantifiable information (Table S2). The error rate of peptides was distributed between −5 and 5 ppm, which suggested that the accuracy of the mass spectrometry data meet the requirement (Fig. 2A). The length of more than 95% peptides was distributed between 7 and 21 amino acids (Fig. 2B), which was in consistence with the properties of tryptic peptides. The percentage of protein with a coverage of (0, 20%) was 69.74% (Fig. 2C). By subcellular localization, 907 PM proteins were identified, of which 758 PM proteins with quantifiable information (Table S2), and a good correlation was observed among the replicates of each treatment group (Fig. S2).

### Al^3+^-induced differentially expressed proteins and enrichment analysis

Differentially expressed proteins (DEPs) in PM of TBS roots between Al^3+^-treated and untreated control groups (CK) were examined. Ninety PM proteins were found to be significant difference in expression in the Al^3+^-treated group, which included 46 up-regulated (FC (fold change) > 1.3, *p* < 0.05) and 44 down-regulated (FC < 0.77, *p* < 0.05) (Table S3).

**Figure 3:**
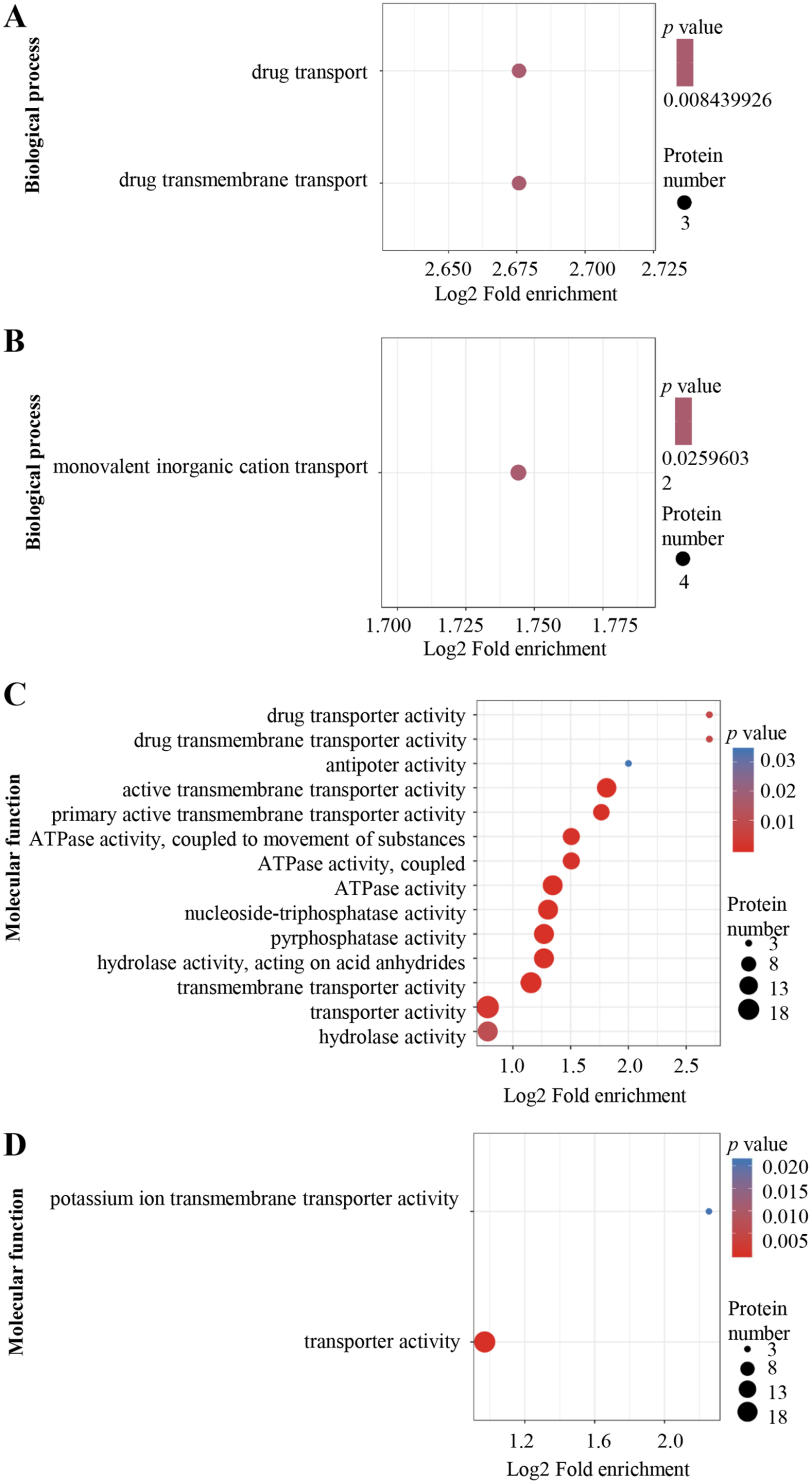
Al^3+^-induced differentially expressed proteins and GO enrichment analysis. GO enrichment of up-regulated (A) and down-regulated (B) proteins in terms of biological process; GO enrichment of up-regulated (C) and down-regulated (D) proteins in terms of molecular function. GO enrichment statistical analysis was performed by Fisher’s exact test. The *X*-axis is folded enrichment; the *Y*-axis is functional classification.

The DEPs were annotated using Gene Ontology (GO) terms based on biological process (BP) and molecular function (MF). For BP, enriched GO terms in the up-regulated proteins were drug transport and drug transmembrane transport (Fig. 3A), whereas those in the down-regulated proteins were mainly associated with monovalent inorganic cation transport process (Fig. 3B). For MF, enriched GO terms in the up-regulated proteins included drug transporter activity, drug transporter transmembrane activity, antiporter activity, active transmembrane transporter activity, primary active transmembrane transporter activity, ATPase activity, nucleoside-triphosphatase activity, pyrophosphatase activity, transporter transmembrane activity, transporter activity and hydrolase activity (Fig. 3C), while the most over-represented GO terms in the down-regulated proteins were transporter activity and potassium ion transmembrane transporter activity (Fig. 3D).

**Figure 4:**
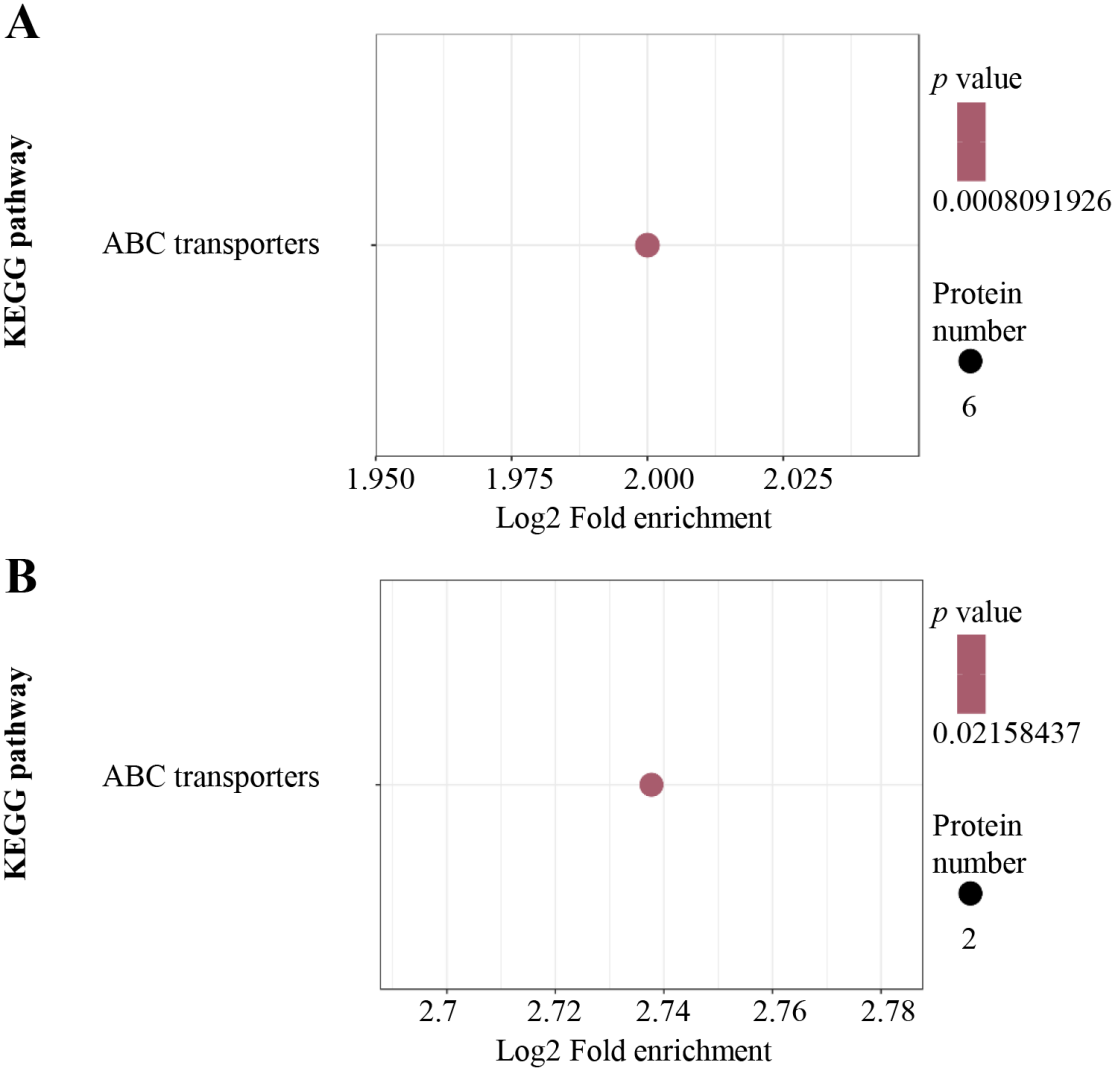
KEGG pathway enrichment analysis. KEGG pathway enrichment of up-regulated (A) and down-regulated (B) proteins. KEGG pathway enrichment statistical analysis was performed by Fisher’s exact test. The *X*-axis is folded enrichment; the *Y*-axis is enrichment pathway.

For KEGG pathway enrichment analysis, ABC transporters were significantly enriched in both the up-regulated (Fig. 4A) and down-regulated (Fig. 4B) DEPs.

**Figure 5:**
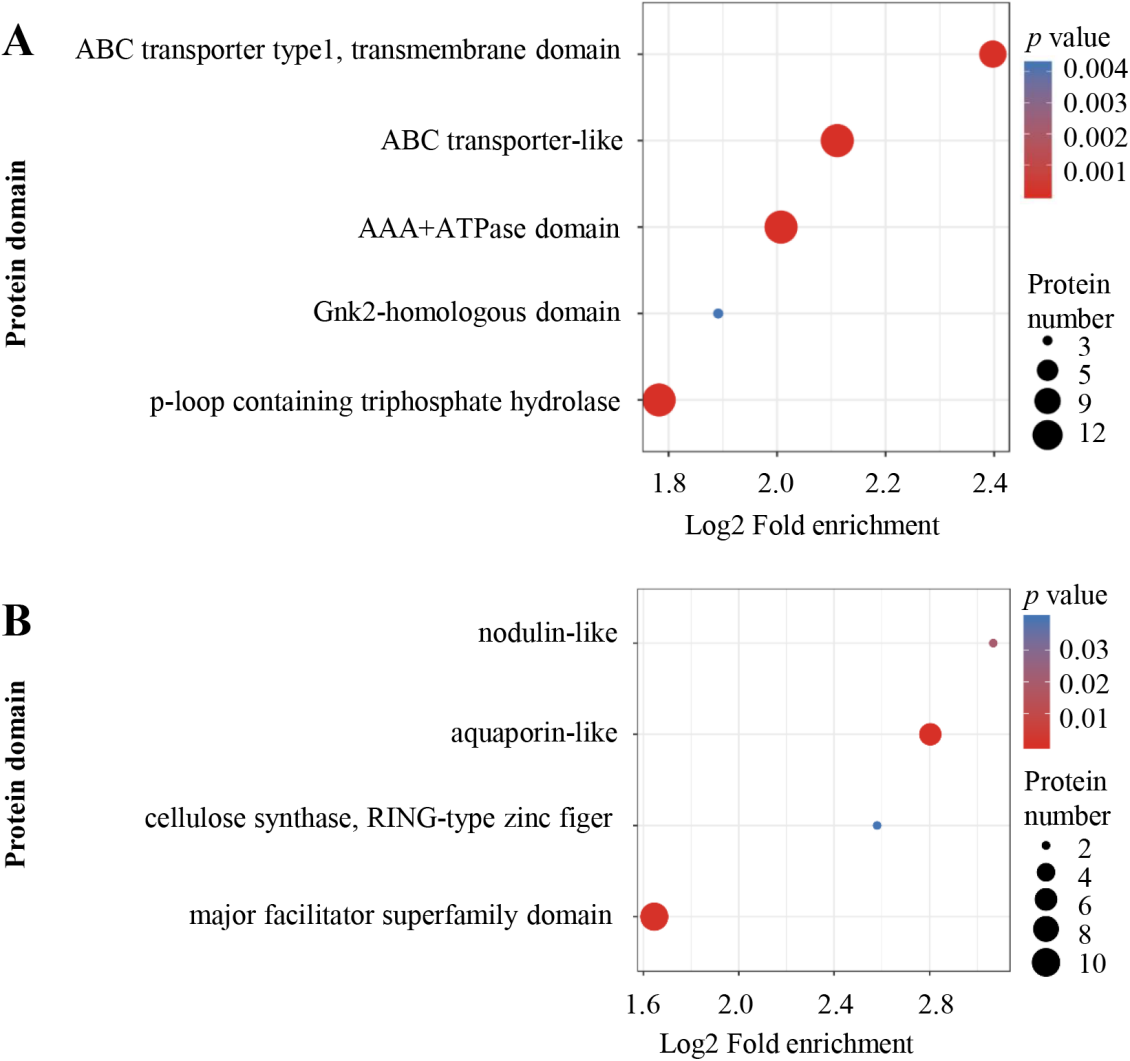
Protein domain enrichment analysis. Enrichment of protein domain analysis in up-regulated (A) and down-regulated (B) proteins. Protein domain enrichment statistical analysis was performed by Fisher’s exact test. The *X*-axis is folded enrichment; the *Y*-axis is protein domain.

Protein domain enrichment analysis was performed at *p* < 0.05 using InterPro database. From high to low, the order of enriched domains in the up-regulated proteins included ABC transporter type 1, ABC transporter-like, AAA+ ATPase domain, P-loop containing nucleoside triphosphate hydrolase and Gnk2 homologous domain (Fig. 5A), whereas the down-regulated proteins contained major facilitator superfamily domain, aquaporin-like, nodulin-like and cellulose synthase (Fig. 5B).

To test the correlation of proteins with different FC changes, DEPs were divided into four Q groups: Q1 (0 < FC ≤ 0.67), Q2 (0.67 < FC ≤ 0.77), Q3 (1.3 < FC ≤1.5) and Q4 (FC >1.5). For each Q group, enrichment and cluster analysis were performed based on GO, KEGG pathway and protein domain. As shown in Figs. S3–S5, the results were consistent with GO, KEGG pathway and protein domain enrichment analysis.

### Validation of TMT data by PRM and RT-qPCR

**Figure 6:**
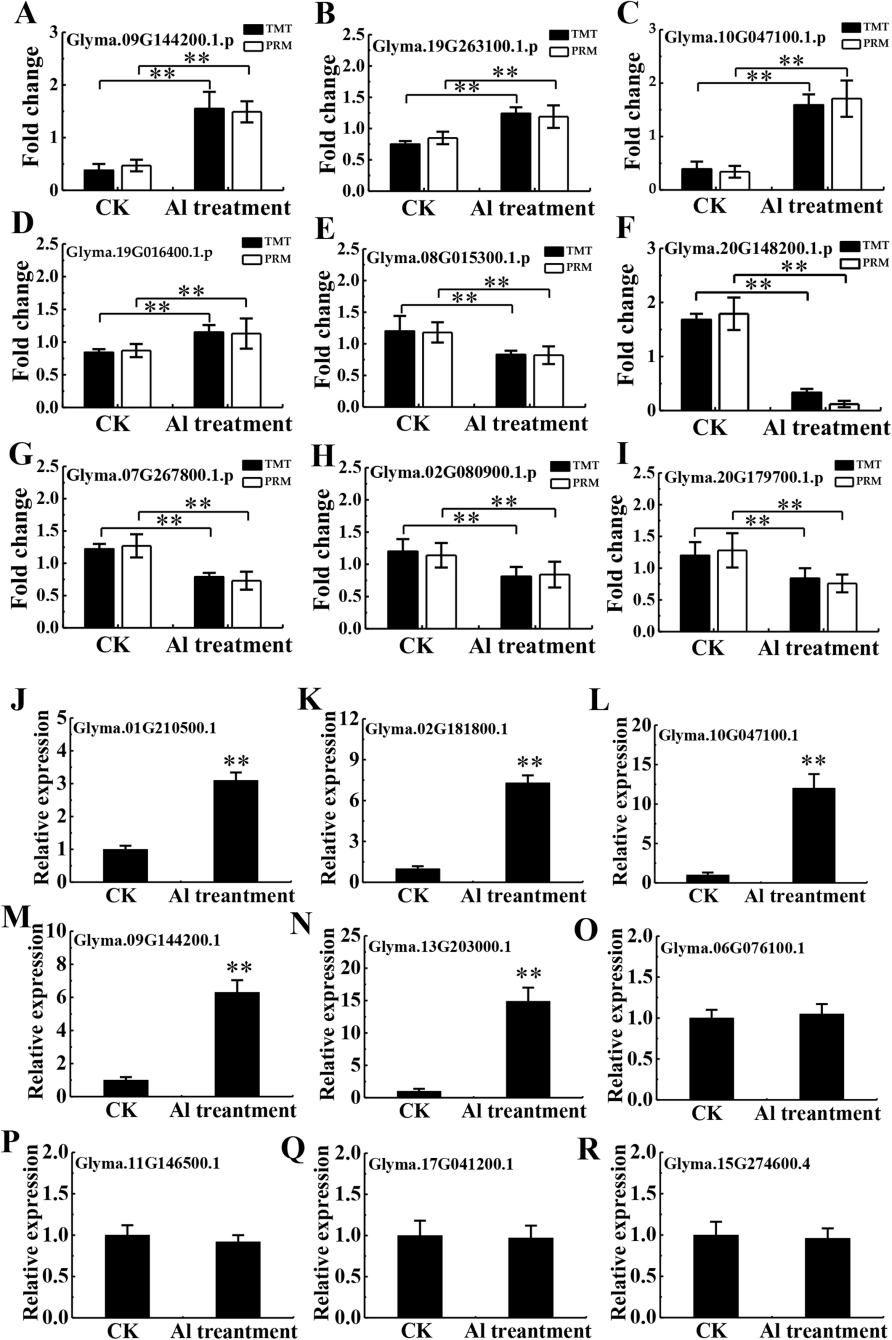
Validation of TMT data by PRM and RT-qPCR. Relative expression levels of selected proteins measured by PRM (A–I) and RT-qPCR analysis of genes encoding the selected proteins (J–R) between the CK and Al treatment in roots of TBS seedlings. CK, the control without Al^3+^ treatment (pH 4.3) for 3 d. Three biological replicates were performed. Two asterisks (**) represent significant differences at *p* < 0.01.

To validate the TMT data, 20 and 9 proteins were randomly chosen for PRM and RT-qPCR, respectively. Of the 20 chosen for PRM analysis, 16 proteins had quantitative information (Table S4), and 9 proteins with a good consistency between TMT and PRM results were shown in Figs. 6A–6I, including the protein abundance of four up-regulated (Glyma.09G144200.1.p, Glyma.19G263100.1.p, Glyma.10G047100.1.p and Glyma.19G016400.1.p) and five down-regulated proteins (Glyma.08G015300.1.p, Glyma.07G267800.1.p, Glyma.02G080900.1.p, Glyma.20G179700.1.p and Glyma.20G148200.1.p). RT-qPCR confirmed enhanced transcription of 5 corresponding genes (Glyma.01G210500.1, Glyma.02G181800.1, Glyma.10G047100.1, Glyma.09G144200.1, Glyma.13G203000.1). However, mRNA expression of 4 related genes (Glyma.06G076100.1, Glyma.11G146500.1, Glyma.17G041200.1, Glyma.15G274600.4) showed no significant difference between Al^3+^-treated and untreated groups (Figs. 6J–6R), differing from the TMT data, which may be due to discrepancy in transcription and post-transcriptional and translational regulation.

### DEPs involved in membrane trafficking and transporters

Fifty-one DEPs involved in membrane trafficking and transporters were identified, of which 29 proteins were up-regulated and 22 proteins were down-regulated by Al stress (Table S5). These proteins mainly included ABC transporters, aquaporins, NRT1, potassium transporters, MATE efflux family proteins, sugar transporters, calcium transporters and H^+^-ATPase. Noticeably, 13 ABC transporters were found to be up-regulated and only one was down-regulated. However, aquaporins, NRT1, potassium and sulfur transporters were found almost to be down-regulated.

### DEPs related to modifying cell wall composition

Five DEPs were related to cell wall synthesis and organization were identified (Table S5), of which cellulose synthases (CESA) (Glyma.04G067900.1.p, Glyma.02G080900.1.p), C2 domain-containing protein (Glyma.11G237800.1.p) and casparian strip membrane protein (CASP) 5 (Glyma.18G290100.1.p) were down-regulated except CASP-like protein (Glyma.20G033800.1.p).

### DEPs of PM associated with defense response

Four DEPs associated with defense response were up-regulated and 2 DEPs were down-regulated (Table S5). The up-regulated DEPs included probable methyltransferase PMT16 (Glyma.09G144200.1.p), MLO-like protein 15 (Glyma.03G093600.2.p), Pleiotropic drug resistance protein 1 (Glyma.07G033500.1.p, Glyma.19G169400.1.p). The down-regulated DEPs comprised probable methyltransferase PMT26 (Glyma.04G211300.1.p) and Cytochrome B561 (Glyma.16G024500.2.p).

### DEPs of PM belonged to signal transduction

**Figure 7:**
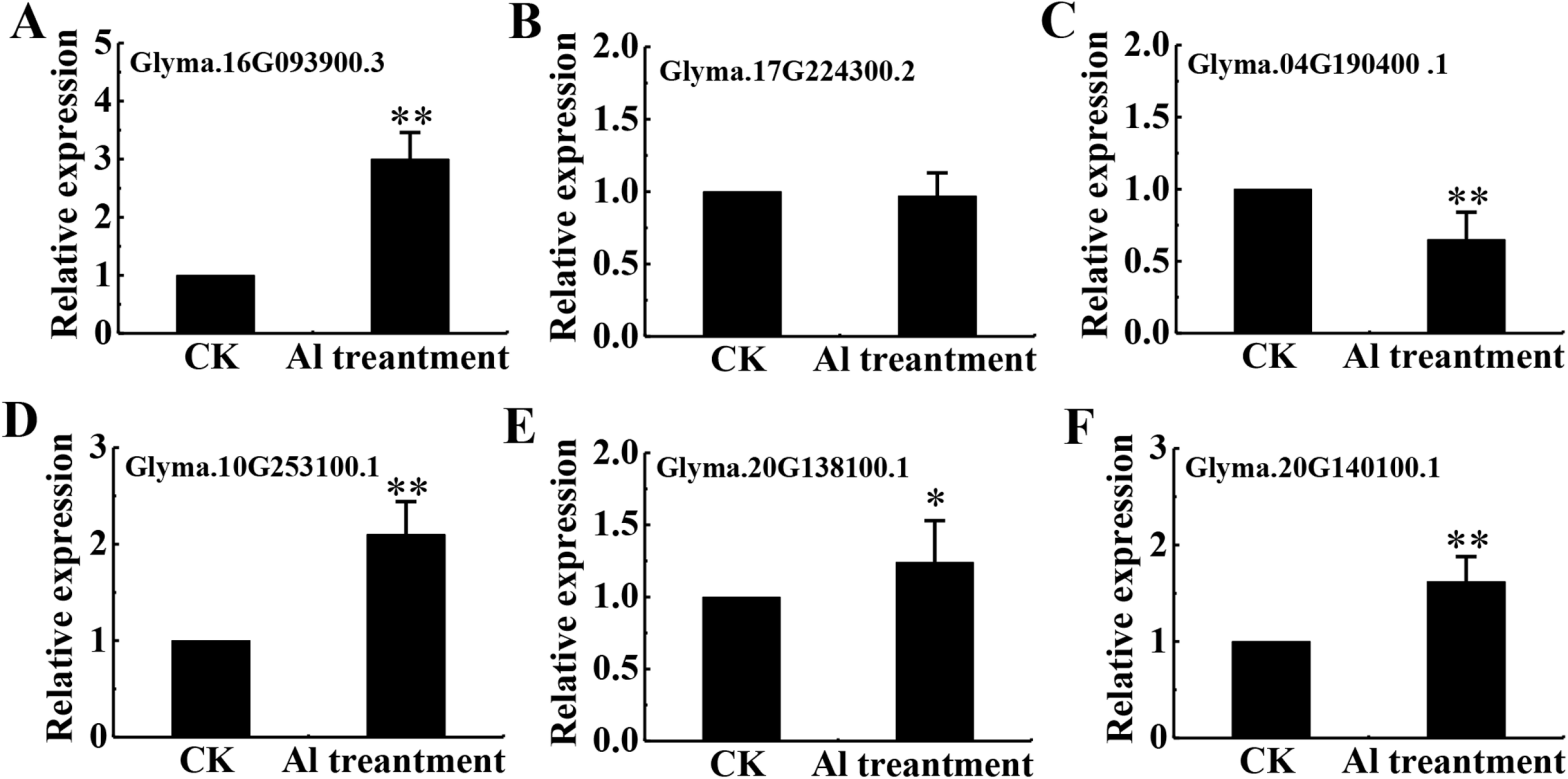
mRNA level of 6 RLKs determined by qRT-PCR. Relative expression levels of 6 RLKs measured by RT-qPCR analysis (A–F) between the CK and Al treatment in roots of TBS seedlings. CK, the control without Al^3+^ treatment (pH 4.3) for 3 d. Three biological replicates were performed. An asterisk (*) represents significant differences at *p* < 0.05, two asterisks (**) represent significant differences at *p* < 0.01.

Eighteen DEPs were belonged to signal transduction with 13 belonging to protein kinases (7 up-regulated and 6 down-regulated), 3 up-regulated associated with Ca^2+^ signaling and 2 down-regulated related to transmembrane receptors (Table S5). Among protein kinases, 6 up-regulated DEPs were classified as receptor kinase and determined by qRT-PCR (Figs. 7A–7F).

## Discussion

In the present study, the optimal time and concentration of Al^3+^ treatment, at which citrate secretion reached its highest level with slight damage in the roots, was determined for PM proteomic analysis (Fig. 1). This makes it possible to get a rich source for investigating the pathways related to Al stress in TBS seedlings. The enrichment of PM proteins were performed by a simplified method and subsequently identified and quantified. The high reproducibility of treatment replicates (Fig. S2) and consistence of peptide error rate and length distribution with the properties of tryptic peptides indicated the accuracy of the mass spectrometry data (Fig. 2), which was further confirmed by PRM and RT-qPCR analyses (Fig. 6). Especially, 90 DEPs, mainly enriched in transporters and signal transduction (Figs. 3–5), provided rich candidate proteins for investigating Al signal perception and tolerance mechanisms in TBS.

### DEPs for Al stress in TBS roots

It has been showed that Al toxicity influenced the uptake of water and nutrients, thereby resulting in the inhibition of plant growth (Sade et al., 2016; Singh et al., 2017). Aquaporins are membrane channels that facilitate the transport of water and small neutral molecules across membrane and the buildup of turgor pressure driving cell elongation (Li et al., 2017). Previous data revealed that Al inhibits the expression of aquaporin genes in buckwheat (Xu et al., 2017), but aquaporin confers Al resistance by mediating Al uptake and translocation in blue hydrangea and Arabidopsis (Negishi et al., 2012; Wang et al., 2017). The decreased abundance of 6 aquaporin proteins found in this study indicates interfere of Al^3+^ with nutrient uptake in TBS roots (Table S5). This result was further supported by expression changes of proteins linked with nitrogen, potassium, sulfur and others transportation.

We also found Al^3+^ affected proteins associated with modifying cell wall composition. Plant cell wall is mainly consisted of cellulose, generated by integral membrane cellulose synthase (CESA) through depositing cellulose polymers into the developing cell wall (Watanabe et al., 2018). For example, *SOS6* encoded a cellulose synthase-like protein in *Arabidopsis*, and *SOS6* mutant results in sensitive to salt-induced osmotic stress due to defect in cell wall formation (Zhu et al., 2010). Indeed, 3 CESAs were markedly reduced under Al^3+^ treatment in TBS membrane (Table S5). Furthermore, 2 Casparian strip membrane proteins (CASPs) were affected by Al^3+^ in this study. CASPs provide the key scaffold for the formation and localization of casparian strips and functions in nutrient uptake and stress resistance (Roppolo et al., 2011), which was supported by the findings that cold and cadmium tolerance in upland cotton and *Arabidopsis* was associated with increased expression of CASPs (Chen et al., 2019). Thus, Al stress-responsive in TBS roots involves cell wall modification via affecting the expression of CESAs and CASPs in plasma membrane, thereby affecting plant root growth.

### DEPs for Al detoxification in TBS roots

**Figure 8:**
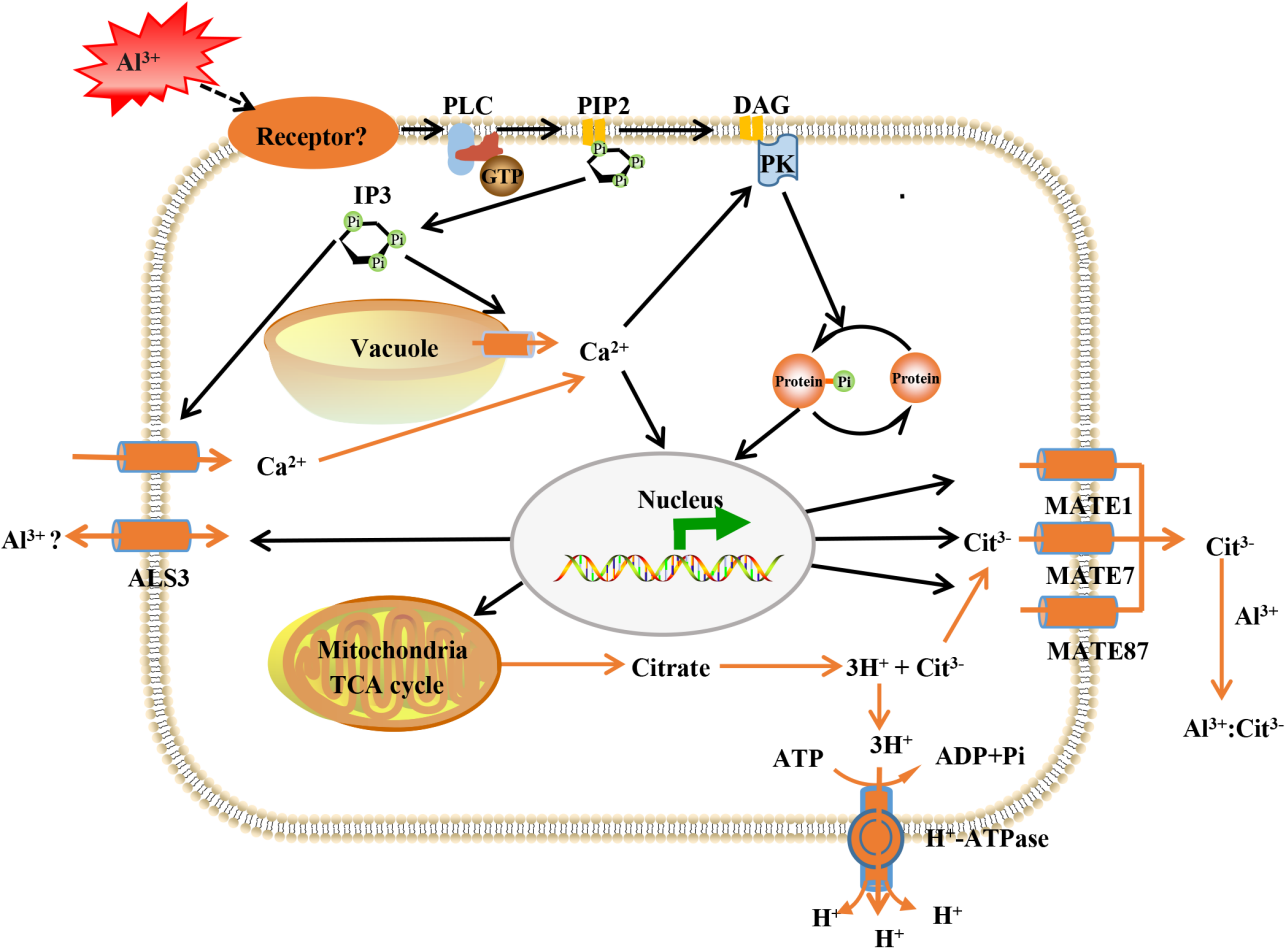
Model illustrating putative aluminum mediated signal transduction and transcriptional regulation pathways in TBS. PLC, phospholipase C; PIP2, phosphatidylinositol diphosphate; IP3, inositol trisphosphate; DAG, diacylglycerol; PK, protein kinases; ALS3, aluminum sensitive protein 3; MATE, multidrug and toxic compound extrusion protein. The black lines represent signal transduction; the yellow line represent transport.

The Al-activated release of citrate from root apex, has been characterized as one of the most important Al-resistance mechanisms in soybean (Kochian et al., 2015), and MATE transporters participate in citrate secretion (Li et al., 2017; Liu et al., 2018). Here, the expression of three MATE proteins (GmMATE13, GmMATE75 and GmMATE87) were up-regulated by Al stress (Table S5). In consistence, overexpression of GmMATE75 and GmMATE87 in root apex or base zone resulted in more citrate efflux, thus alleviated root elongation inhibition by Al^3+^ in Arabidopsis (Zhou et al., 2019). Similarly, GmMATE13 has been confirmed to mediate citrate exudation and then to enhance Al resistance in soybean hairy roots (Wang et al., 2019a, 2019c). In addition, up-regulation of H^+^-ATPase was found under Al stress (Table S5). PM H^+^-ATPase extrudes protons from cells to create an electrochemical gradient across the plasma membrane that enables the release of citrate under Al toxicity and phosphorus deficiency (Yu et al., 2016). Our previous data indicated that Al enhanced the expression the PM H^+^-ATPase and interaction between PM H^+^-ATPase and the 14-3-3 protein led to higher activity of the PM H^+^-ATPase and more citrate exudation in broad bean (Chen et al., 2013). Although further investigations are needed, these results lead us to conclude that GmMATE13, GmMATE75, GmMATE87 and H^+^-ATPase collectively function in Al resistance via releasing citrate in TBS roots (Fig. 8).

On the other hand, most ABC transporters are present in cell membranes and are essential for plant nutrition, hormone transport, pathogen responses and lipid deposition (Kochian et al., 2015; Wang et al., 2019b). ABC transporters have been implicated in the tolerance to metal toxicity (Ezaki et al., 2015), such as cadmium in rice (Fu et al., 2019). Accordingly, Al^3+^ induced twelve ABC transporters in the present study, including 2 ALS3 proteins and 5 ABCB transporters (Table S5). ALS3 functions in redistributing Al in plants away from the sensitive root tips, and transport Al into vacuole for detoxification (Larsen et al., 2005; Reuna-Llorens et al., 2014). Furthermore, ALS3 interacts with its functional partner sensitive to Al rhizotoxicity 1 (STAR1), and STAR1-STAR2 complex transport UDP-glucose into the cell wall, thereby masking Al-binding in the cell wall (Kochian et al., 2015). In addition, ABCB transporters participate in the transport of auxin, which enhances Al-induced citrate exudation through up-regulation of GmMATE and activation of the plasma membrane H^+^-ATPase in TBS roots in our previous study (Wang et al., 2016). Thus, the expression patterns of above important transporters indicated that TBS harbors avoidance and tolerance strategies for Al-detoxification.

### DEPs for defense responses in TBS roots

Evidence shows methyltransferases decreasing ROS, lipid peroxidation and ion leakage, and heterologous expression of methyltransferase in *Escherichia coli* confers Al resistance through melatonin production (Luo, He & Han, 2018). Likewise, pleiotropic drug resistance (PDR) proteins confer the plant to have the capacity of stress response and cellular detoxification (Wolfger, Mamnun & Kuchler, 2001). NtPDR1 is involved in the general defense response in tobacco (Sasabe et al., 2002). It showed that the silence of NpPDR1 resulted in an increased susceptibility to sclareol and a reduced resistance to plant pathogen (Stukkens et al., 2005). Additionally, MLO proteins play a broad role in plants, such as stress response processes and cell death protection (Kim et al., 2002; Stein & Somerville, 2002). MLO-mediated defense suppression functions independently of heterotrimeric G-proteins, and functions in Ca^2+^-dependent interaction with calmodulin (Kim et al., 2002). The enhanced abundance of PMT16, PDR1 and MLO15 (Table S5) indicated their involvement in Al^3+^ resistance in TBS.

### DEPs for signal transduction

We previously revealed that Al^3+^ signal transmits through heterotrimeric G-proteins, PLC, IP3, DAG, Ca^2+^ and PK, which result in citrate secretion from roots via modifying tricarboxylic cycle (Jiang et al., 2018) (Fig. 8). Recently, Al receptors or sensors have been put forward, and identification of the upstream elements for sensing extracellular Al signal remains to be an open and important question in plant Al stress research. The plasma membrane is the first site of signal perception through receptor proteins and membrane receptors for Al^3+^ perception has been recognized (Yang, Fan & Zheng, 2019). Particularly, it has been established that plant receptor-like kinases (RLKs) act as receptors for extracellular signal perception in abiotic stress responses (Morris & Walker, 2003; Ye et al., 2017). Accordingly, many RLKs have been identified for stress signal detection, such as CPKs for salt sensing (Gao et al., 2018), CRK5 for drought sensing (Burdiak et al., 2015), RLCK for *Pseudomonas syringae* sensing (Liu et al., 2019). As expected, 6 RLKs were found to be enhanced in expression after Al^3+^ treatment (Table S5). This result provided candidate proteins for further investigation on the upstream signal element of Al signaling in TBS.

Calcium ion (Ca^2+^) serves as an essential second messenger to regulate many cellular activities, thereby achieving a specific physiological response (Yang et al., 2019a, 2019b). Our previous data also established that cytoplasmic Ca^2+^, an essential intracellular second messenger, mediates Al signaling in stylo (Jiang et al., 2018). Calcium channel proteins are critical regulators of calcium homeostasis, and calcium-binding proteins can activate many calcium dependent protein kinases (Chen et al., 2015). Disruption of intracellular Ca^2+^ upon Al^3+^ exposure triggers a significant decrease of citrate release in the root (Chen et al., 2015; Jiang et al., 2018). In this research, abundance of 5 proteins associated calcium signaling were up-regulated under Al stress, including 2 calcium channel proteins, 2 calcium-binding proteins and 1 calcium-dependent lipid-binding family protein (Table S5). These results lend supports to the involvement of Ca^2+^ in Al-induced citrate exudation in TBS.

## Conclusions

In this study, TMT-based quantitative PM proteome was used to explore the mechanism of TBS Al resistance. The 907 PM proteins were identified, of which 90 proteins were considered as DEPs with 46 up-regulated and 44 down-regulated. These DEPs mainly involved in membrane trafficking and transporters, modifying cell wall composition, defense responses and signal transduction. Our results indicated that GmMATE13, GmMATE75, GmMATE87 and H^+^-ATPase were involved in Al-induced exudation of citrate in PM of TBS roots, and ABC transporters and Ca^2+^ have been implicated in internal detoxification and signaling of Al, respectively. Importantly, our data provides 6 RLKs as candidate proteins for further investigating Al signal transmembrane mechanisms.

## Supplemental Information

10.7717/peerj.9312/supp-1 Supplemental Information 1 Strategy of analysis of PM protein expression in in TBS by 6-plex isobaric tagging. 126, 127 and 128 represent three samples of the control group, 129, 130 and 131 represent three samples of the Al-treated group.

10.7717/peerj.9312/supp-2 Supplemental Information 2 Repeatability test between PM proteins of samples. C1, C2 and C3 represent three samples of the control group, A1, A2 and A3 represent three samples of the Al-treated group.

10.7717/peerj.9312/supp-3 Supplemental Information 3 GO functional cluster of DEPs in CK and Al treatment. (A) GO functional cluster of DEPs in the biological process. (B) GO functional cluster of DEPs in the molecular function.

10.7717/peerj.9312/supp-4 Supplemental Information 4 Cluster analysis of DEPs based on KEGG pathway.

10.7717/peerj.9312/supp-5 Supplemental Information 5 Cluster analysis of DEPs based on protein domain.

10.7717/peerj.9312/supp-6 Supplemental Information 6 Primer sequences used in this study.

10.7717/peerj.9312/supp-7 Supplemental Information 7 All identified proteins in TBS roots.

10.7717/peerj.9312/supp-8 Supplemental Information 8 Differentially regulated proteins in PM of TBS roots between Al3+-treated and untreated control groups.

10.7717/peerj.9312/supp-9 Supplemental Information 9 Protein quantitative information by PRM. The proteins numbered 1-9 with a good consistency between TMT and PRM results appear in the article.

10.7717/peerj.9312/supp-10 Supplemental Information 10 Functional classification of PM DEPs.

10.7717/peerj.9312/supp-11 Supplemental Information 11 RT-qPCR data to reproduce (Fig 6).

10.7717/peerj.9312/supp-12 Supplemental Information 12 Citrate secretion (Fig 1A and 1B).

## Additional Information and Declarations

### Data Availability

The following information was supplied regarding data availability: The data is available at ProteomeXchange via the PRIDE partner repository: PXD017160.
