# Supplementary figures and images for: Plasma membrane proteomic analysis by TMT-PRM provides insight into mechanisms of aluminum resistance in tamba black soybean roots tips
Source: PeerJ. 2020 Jun 10;8:e9312. doi: 10.7717/peerj.9312 (PMC7293186; doi:10.7717/peerj.9312)

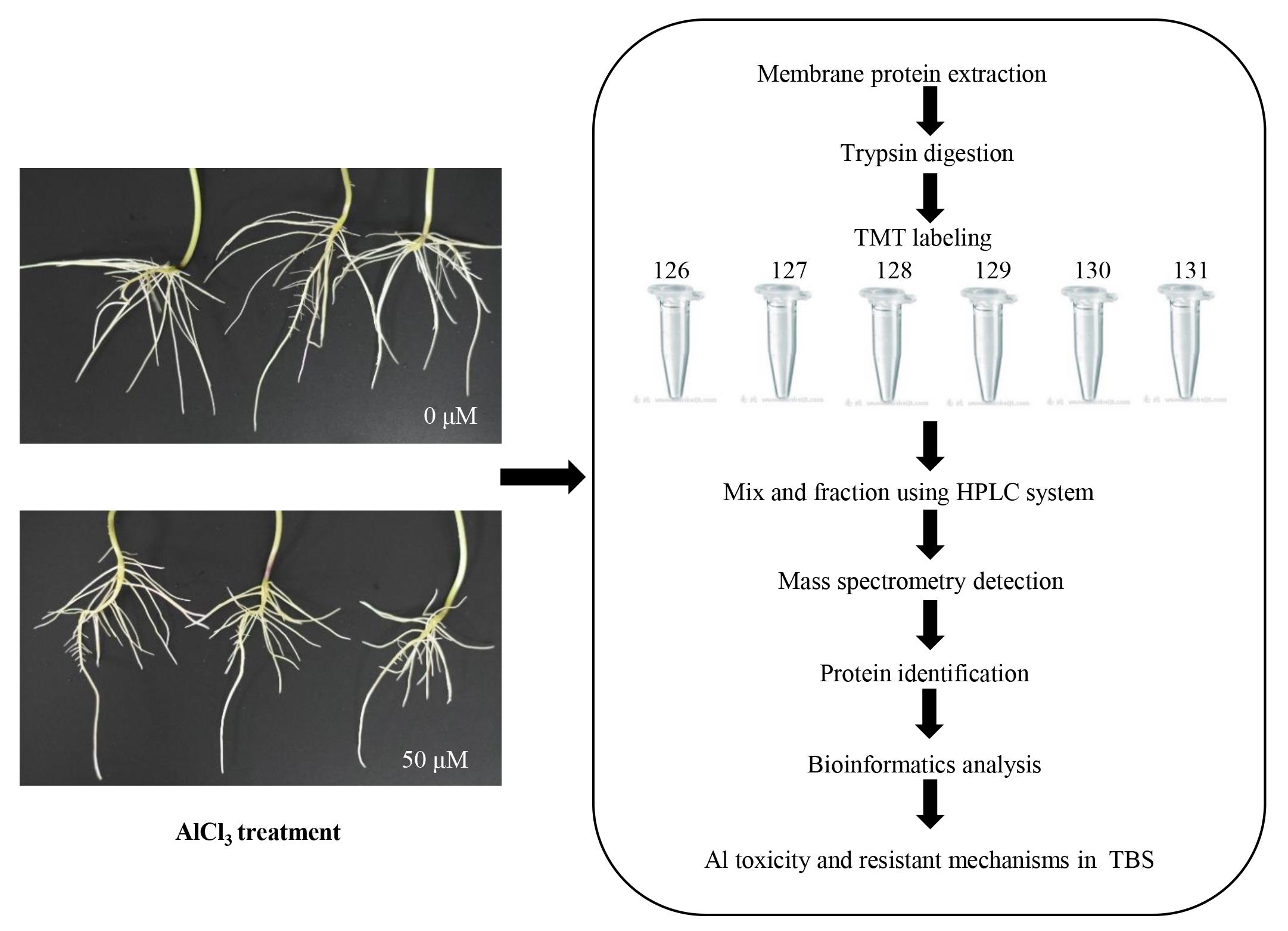

Supplement: Supplemental Information 1 — 126, 127 and 128 represent three samples of the control group, 129, 130 and 131 represent three samples of the Al-treated group. [file peerj-08-9312-s001.png]

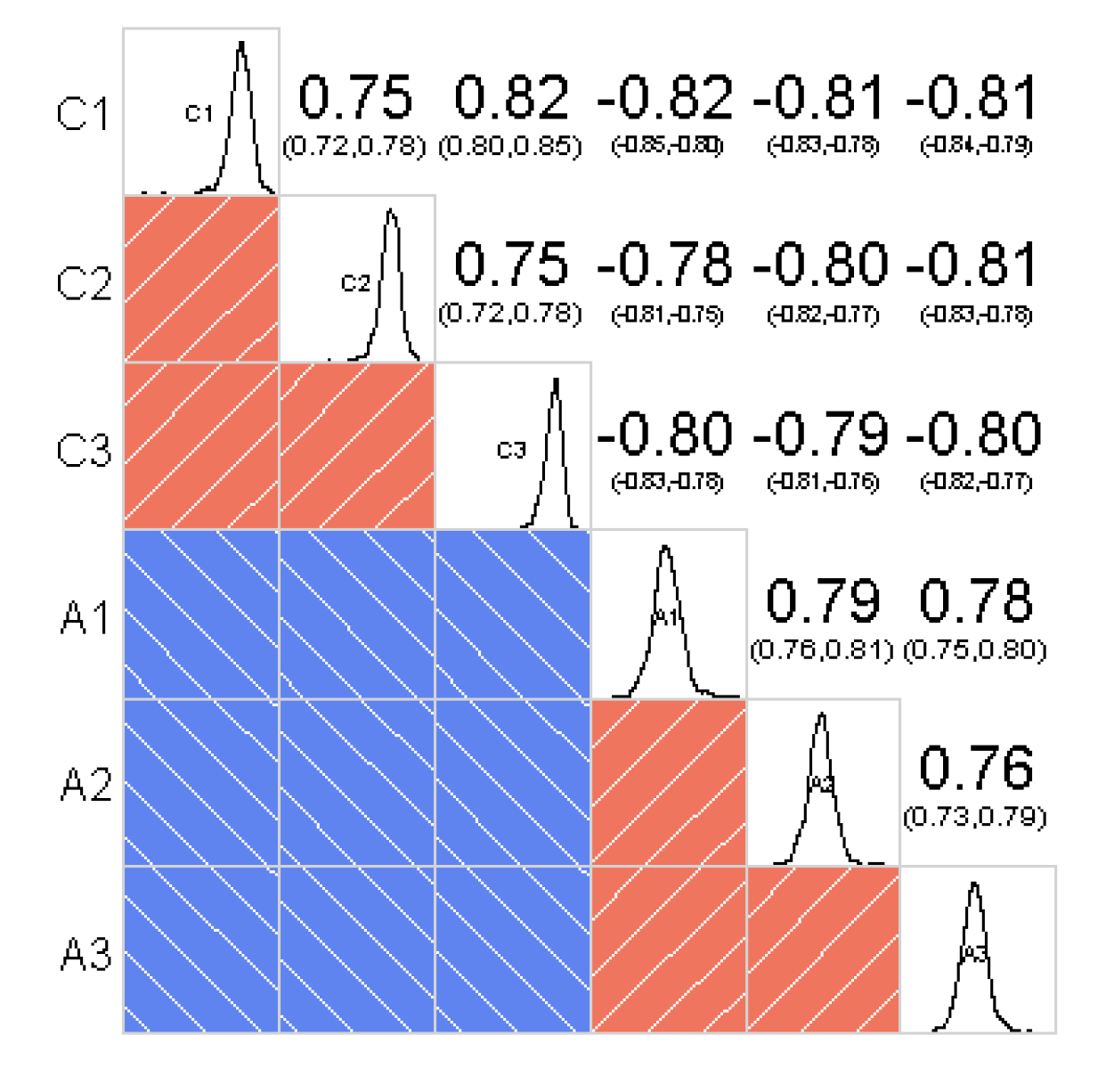

Supplement: Supplemental Information 2 — C1, C2 and C3 represent three samples of the control group, A1, A2 and A3 represent three samples of the Al-treated group. [file peerj-08-9312-s002.png]

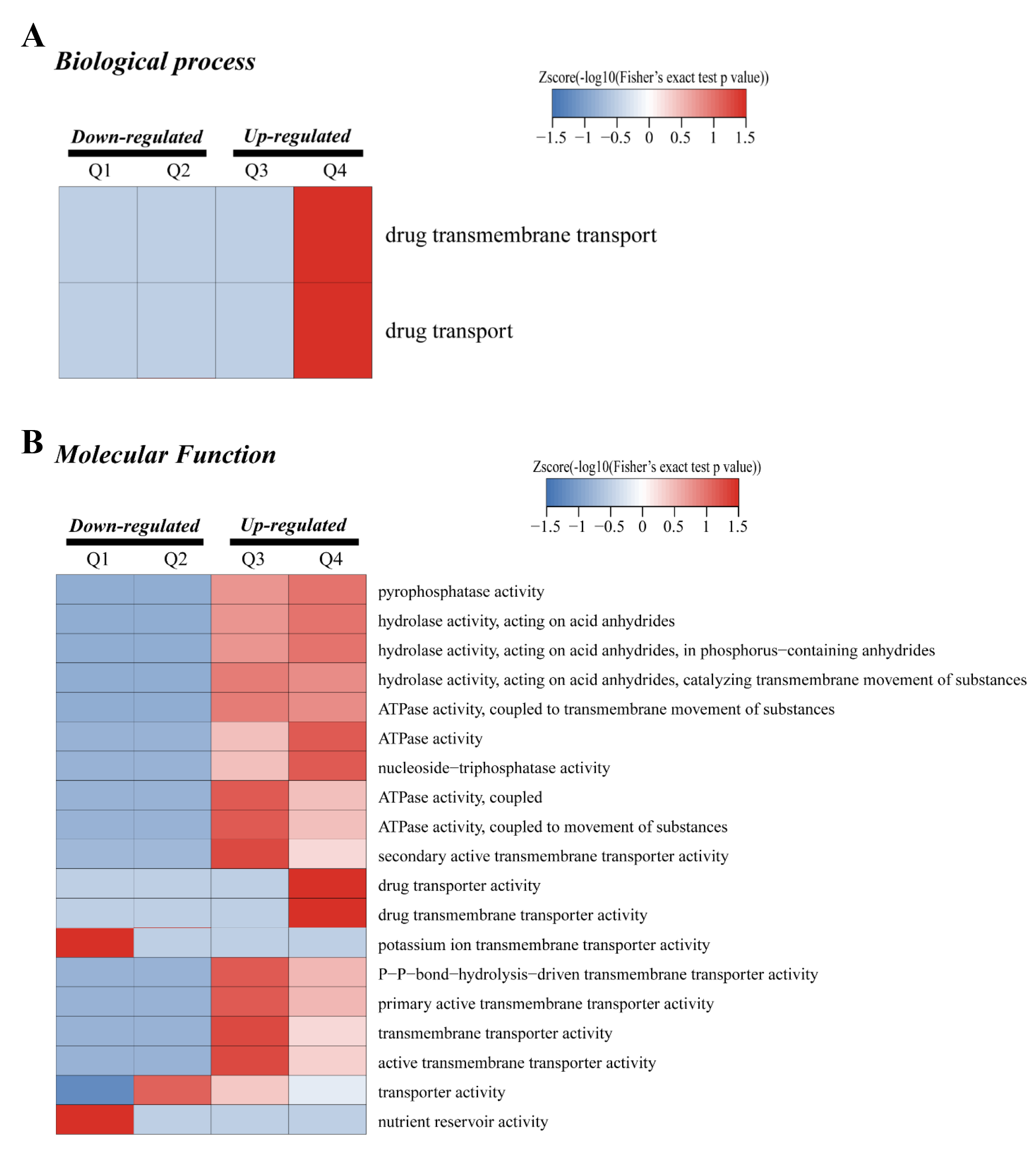

Supplement: Supplemental Information 3 — (A) GO functional cluster of DEPs in the biological process. (B) GO functional cluster of DEPs in the molecular function. [file peerj-08-9312-s003.png]

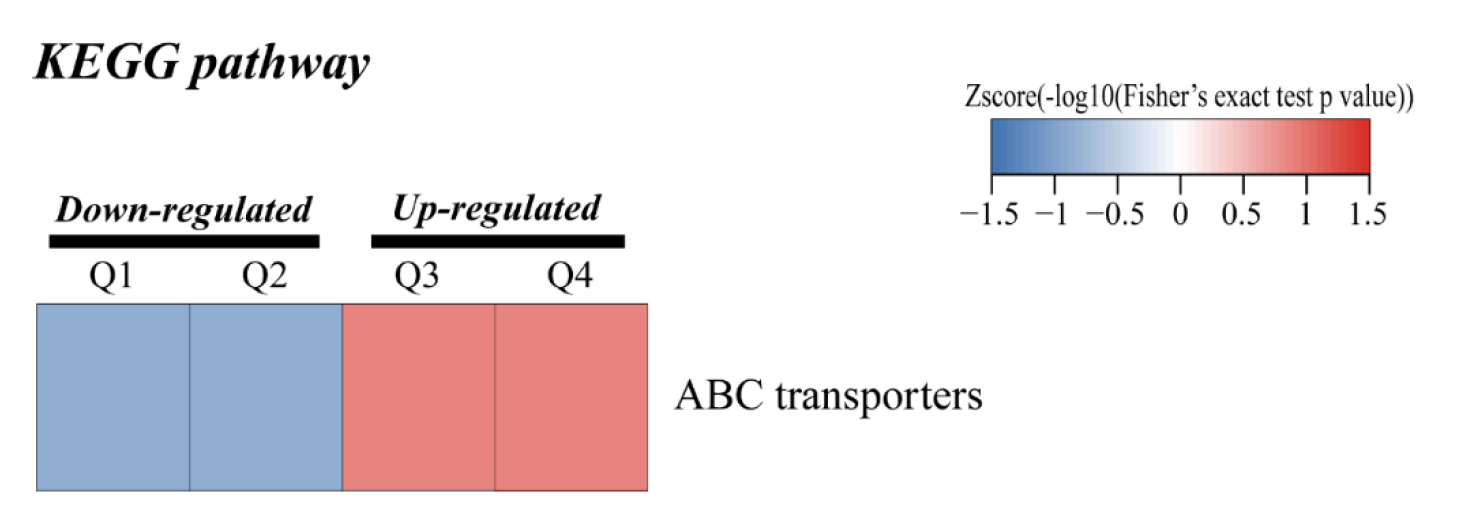

Supplement: Supplemental Information 4 [file peerj-08-9312-s004.png]

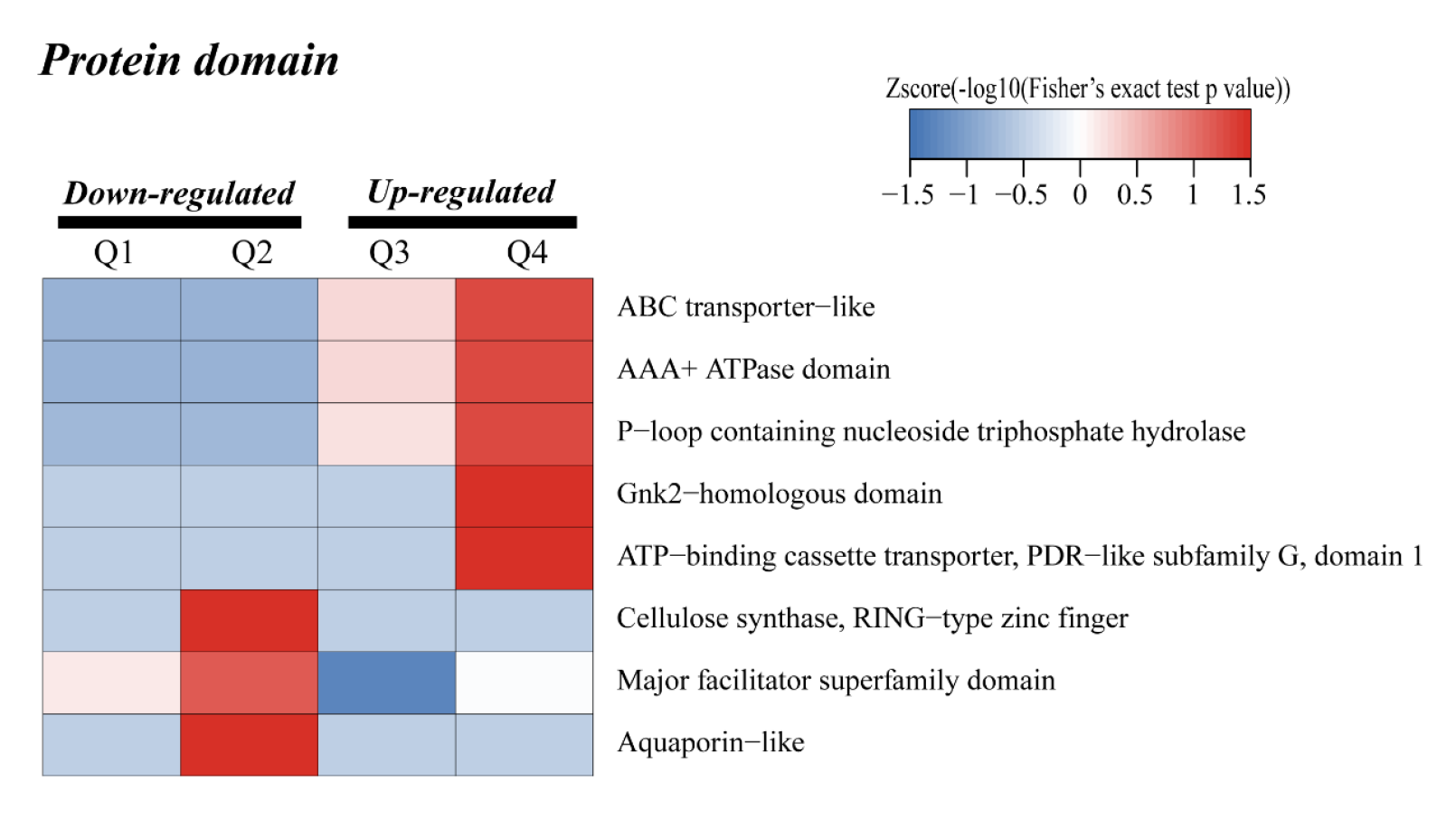

Supplement: Supplemental Information 5 [file peerj-08-9312-s005.png]
